# Magnetic resonance elastography for the prediction of hepatocellular carcinoma in chronic hepatitis B

**DOI:** 10.1002/jgh3.13067

**Published:** 2024-04-25

**Authors:** Nobuharu Tamaki, Mayu Higuchi, Taisei Keitoku, Yudai Yamazaki, Naoki Uchihara, Keito Suzuki, Yuki Tanaka, Haruka Miyamoto, Michiko Yamada, Risa Okada, Kenta Takaura, Shohei Tanaka, Chiaki Maeyashiki, Yutaka Yasui, Kaoru Tsuchiya, Hiroyuki Nakanishi, Tatsuya Kanto, Masayuki Kurosaki, Namiki Izumi

**Affiliations:** ^1^ Department of Gastroenterology and Hepatology Musashino Red Cross Hospital Tokyo Japan; ^2^ Department of Liver Disease The Research Center for Hepatitis and Immunology, National Center for Global Health and Medicine Chiba Japan

**Keywords:** chronic hepatitis B, hepatocellular carcinoma (HCC), liver fibrosis, magnetic resonance elastography (MRE)

## Abstract

**Background and Aim:**

Magnetic resonance elastography (MRE) is used for the evaluation of liver fibrosis; however, it remains unclear whether MRE‐based liver stiffness is associated with hepatocellular carcinoma (HCC) development, particularly in patients with chronic hepatitis B.

**Methods:**

A total of 504 patients with chronic hepatitis B receiving MRE were enrolled. The *primary endpoint* was the association between MRE‐based liver stiffness and HCC.

**Results:**

In a cross‐sectional analysis at the time of MRE measurement, the median (interquartile range) liver stiffness values in patients with presence or history of HCC and those without HCC were 3.68 (2.89–4.96) and 2.60 (2.22–3.45) kPa, respectively, and liver stiffness was significantly higher in patients with presence or history of HCC than in those without HCC (*P* < 0.001). In a longitudinal analysis of patients without HCC, the 1‐, 3‐, and 5‐year cumulative incidence of HCC in patients with liver stiffness ≥3.6 kPa and those with liver stiffness <3.6 kPa were 3.8%, 7.0%, and 22.9%, and 0%, 0.9%, and 1.5%, respectively (*P* < 0.001). In the multivariable analysis, MRE‐based liver stiffness (per 1 kPa) or liver stiffness ≥3.6 kPa was an independent factor for HCC development with an adjusted hazard ratio (aHR) of 1.61 (95% confidence interval [CI], 1.3–2.0) or aHR of 8.22 (95% CI, 2.1–31).

**Conclusion:**

MRE‐based liver stiffness is associated with HCC risk in patients with chronic hepatitis B and may be used for the early prediction of HCC development and determination of indications for treatment.

## Graphical abstract

MRE‐based liver stiffness is associated with HCC risk in patients with chronic hepatitis B and may be used for the early prediction of HCC development and determination of indicators for treatment.

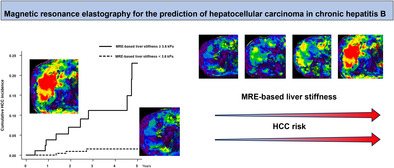

## Introduction

Chronic hepatitis B is one of the most important liver diseases worldwide, in which approximately 300 million people were infected with the hepatitis B virus.^1^, ^2^ Chronic hepatitis B progresses to hepatocellular carcinoma (HCC).^3^ Moreover, the prevention and early detection of HCC due to chronic hepatitis B is an important clinical issue.^4^

Liver fibrosis is the most important factor associated with HCC development in patients with chronic hepatitis, including hepatitis B.^5^, ^6^ Therefore, accurate assessment of liver fibrosis status is crucial in clinical practice. Although liver biopsy is the gold standard for assessing liver fibrosis, liver biopsy has several limitations including invasiveness, complications, and cost.^7^ To overcome these limitations, several noninvasive methods for estimating liver features including fibrosis have been developed and used in clinical practice.^8^, ^9^, ^10^, ^11^, ^12^

Magnetic resonance elastography (MRE) is a noninvasive method for estimating liver fibrosis. Previous studies have demonstrated that MRE‐based liver stiffness is significantly associated with biopsy‐based liver fibrosis stage and has higher diagnostic accuracy than other noninvasive methods including ultrasound elastography or blood‐based tests.^13^, ^14^ However, data on MRE and HCC development are limited, particularly in patients with chronic hepatitis B, and whether MRE could be used to predict HCC remains unclear. To fill the current gap in knowledge, this study investigated the association between MRE‐based liver stiffness and HCC development in patients with chronic hepatitis B.

## Methods

### Study design

This single‐center, retrospective cohort study included patients with chronic hepatitis B who received MRE at Musashino Red Cross Hospital between January 2015 and August 2022. A total of 530 consecutive patients with chronic hepatitis B who received MRE were investigated. The exclusion criteria are as follows: (1) co‐infection of hepatitis C virus or human immunodeficiency virus, (2) complication of other liver diseases including autoimmune hepatitis or primary biliary cholangitis, and (3) positivity only for hepatitis B core antibodies. Finally, a total of 504 patients were enrolled in the study.

Informed consent was obtained from all patients through an opt‐out method. The study methods conformed to the ethical guidelines of the Declaration of Helsinki, and the study was approved by the institutional ethics review committee.

### Clinical and laboratory data

The index date was the date of MRE measurement. Patient characteristics and laboratory data were collected within 6 months of MRE assessment. Information on age, sex, and comorbidities (diabetes mellitus [DM] and dyslipidemia) were recorded, and standard blood count and biochemistry tests were conducted.

### MRE assessment

MRE was performed using Signa HDxt 1.5T (GE Medical Systems, Waukesha, WI, USA) and MR Touch (GE Healthcare) as previously described.^15^ Briefly, shear waves were generated by external vibration at 60 Hz using a passive driver as the vibration device was slightly placed to the right, lateral to the xiphoid process. Cross‐sectional elastography images of the stiffness generated from the wave propagation information obtained using the gradient echo sequence were obtained. The region of interest was placed at the right hepatic lobe on each slice of the stiffness map, carefully avoiding the liver surface, liver edge, gallbladder, blood vessels, bile ducts, tumors, and artifacts. The mean stiffness value of three regions of interest placed at different slices was used for the analysis. The MRE‐based liver stiffness of 3.6 kPa was used as a threshold for advanced fibrosis based on previous studies.^16^, ^17^

### HCC surveillance and diagnosis

Ultrasonography and blood tests, including tumor marker tests, were performed every 3–6 months for HCC surveillance. When tumor marker levels rose abnormally and/or abdominal ultrasonography suggested a lesion suspicious of HCC, contrast‐enhanced computed tomography, magnetic resonance imaging, or angiography was performed. HCC was diagnosed for tumors displaying vascular enhancement at the early phase and washout at the later phase, according to the guidelines by the American Association for the Study of Liver Diseases and the Japan Society of Hepatology.^18^, ^19^ Tumor biopsy was used to diagnose tumors with nontypical imaging findings.

### Primary endpoint

**Figure 1:**
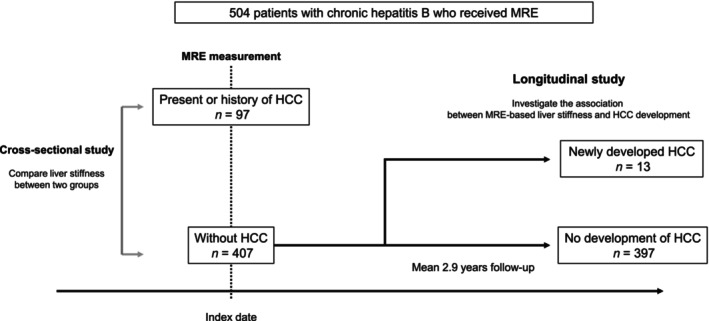
Study protocol.

The primary endpoint was the association between MRE‐based liver stiffness and HCC. In a cross‐sectional analysis, the association between MRE‐based liver stiffness and the presence or history of HCC at the index date (date at MRE measurement) was investigated. In a longitudinal analysis, the cumulative incidence of HCC development was investigated in patients without a history of HCC. The study protocol is shown in Figure 1.

### Statistical analyses

In the cross‐sectional analysis, MRE‐based liver stiffness between patients with HCC or history of HCC and those without HCC was compared using the Mann–Whitney *U* test. In the longitudinal analysis, cumulative incidences of HCC development were calculated using the Kaplan–Meier method and log‐rank test. The factors associated with HCC development were analyzed using the logistic regression model in the cross‐sectional analysis and the Cox‐proportional hazard model in the longitudinal analysis. In the multivariable analyses, age, sex, DM, dyslipidemia, and nucleoside/nucleotide analog (NA) treatment were selected a priori as covariables for HCC development. Statistical significance was defined as *P*‐values of <0.05. All statistical analyses were performed using EZR (Saitama Medical Center, Jichi Medical University, Shimotsuke, Japan), a graphical user interface for R version 3.2.2 (The R Foundation for Statistical Computing, Vienna, Austria).

## Results

### Patient characteristics

A total of 504 patients with chronic hepatitis B whose MRE was measured were enrolled in the study. Patient characteristics are shown in Table 1. The median age (interquartile range [IQR]) was 60 (49–68) years, and 306 (60.7%) of the patients were male. NA therapy was administered in 268 (53.2%) patients. DM and dyslipidemia were 47 (9.3%) and 103 (20.4%), respectively. Regarding HBV status, 21.9% of patients were HBe antigen positive and the median (IQR) HBs antigen was 772 (121–3318) IU/mL. The median (IQR) MRE‐based liver stiffness was 2.76 (2.28–3.70) kPa.

**Table 1:** Patient characteristics

	*n* = 504
Age, years	60 (49–68)
Males, %	306 (60.7%)
Diabetes mellitus, %	47 (9.3%)
Dyslipidemia, %	103 (20.4%)
NA treatment	268 (53.2%)
Albumin, g/dL	4.3 (4.1–4.5)
AST, IU/L	26 (22–35)
ALT, IU/L	23 (16–37)
Platelet counts, 10^9^/L	175 (142–216)
Liver stiffness, kPa	2.76 (2.28–3.70)
HBe antigen, %^†^	
Positive	109 (21.9%)
Negative	388 (78.1%)
HBs antigen, IU/mL	772 (121–3318)

^†^ Data were missing in seven patients.

Continuous data are shown in median (interquartile range).

ALT, alanine aminotransferase; AST, aspartate aminotransferase; NA, nucleoside/nucleotide analog.

### Liver stiffness and HCC in the cross‐sectional study

**Figure 2:**
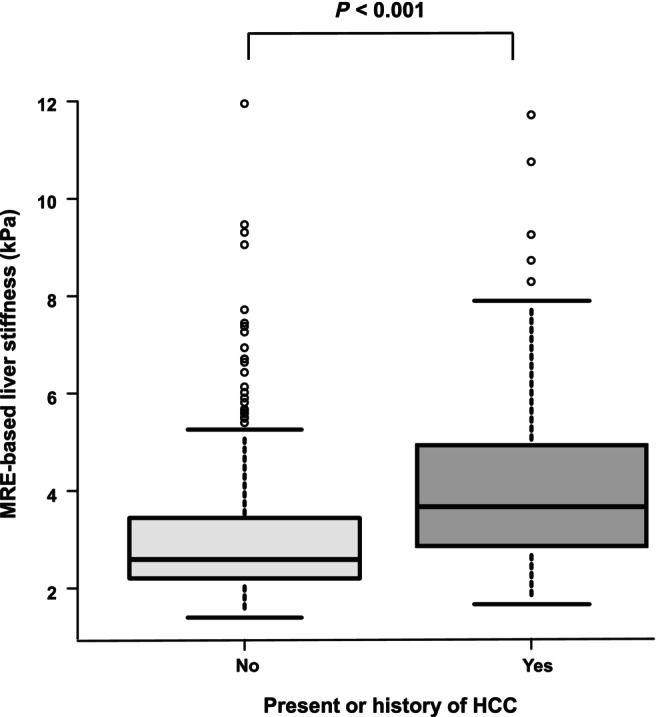
MRE‐based liver stiffness and HCC status at the time of MRE measurement. MRE‐based liver stiffness at the time of MRE measurement was compared between patients with HCC or a history of HCC and those without HCC. The bottom and top of each box represent the 25th and 75th percentiles, giving the interquartile range. The line through the box indicates the median value, and the error bars indicate the 10th and 90th percentiles. HCC hepatocellular carcinoma; MRE, magnetic resonance elastography.

At the time of MRE measurement, 97 patients had HCC or a history of HCC, and 407 patients had no history of HCC. When comparing MRE‐based liver stiffness, the median (IQR) liver stiffness values in patients with HCC or a history of HCC and those without HCC were 3.68 (2.89–4.96) and 2.60 (2.22–3.45) kPa, respectively. Liver stiffness was significantly higher in patients with HCC or a history of HCC than in those without HCC (*P* < 0.001, Fig. 2, Table 2). In addition, patients with HCC or a history of HCC were significantly older and more likely to be male and to have DM. HCC‐associated factors were evaluated in the cross‐sectional analysis. After adjusting for age, sex, NA therapy, DM, and dyslipidemia, MRE‐based liver stiffness (per 1 kPa) was an independent factor for HCC with an odds ratio of 1.30 (95% confidence interval [CI], 1.1–1.5, *P* < 0.001).

**Table 2:** Comparison between patients without HCC and those with the presence or history of HCC in the cross‐sectional study

	Patients without HCC	Patients with the presence or history of HCC	*P* value
	*n* = 407	*n* = 97	
Age, years	58 (48–68)	65 (57–73)	<0.001
Males, %	234 (57.5%)	72 (74.2)	0.003
Diabetes mellitus, %	27 (6.7%)	20 (20.6%)	<0.001
Dyslipidemia, %	89 (21.9%)	14 (14.4%)	0.1
NA treatment	179 (44.0%)	89 (91.8%)	<0.001
Albumin, g/dL	4.3 (4.1–4.5)	4.3 (4.0–4.5)	0.2
AST, IU/L	26 (22–35)	26 (23–34)	0.3
ALT, IU/L	24 (16–40)	22 (16–30)	0.2
Platelet counts, 10^9^/L	182 (150–222)	146 (117–177)	<0.001
Liver stiffness, kPa	2.60 (2.22–3.45)	3.68 (2.89–4.96)	<0.001
HBe antigen, %^†^			
Positive	90 (22.4%)	19 (19.8%)	0.7
HBs antigen, IU/mL	903 (161–4370)	476 (67–1070)	<0.001

^†^ Data were missing in seven patients.

Continuous data are shown in median (interquartile range).

ALT, alanine aminotransferase; AST, aspartate aminotransferase; NA, nucleoside/nucleotide analog.

### Liver stiffness and HCC development in the longitudinal study

**Figure 3:**
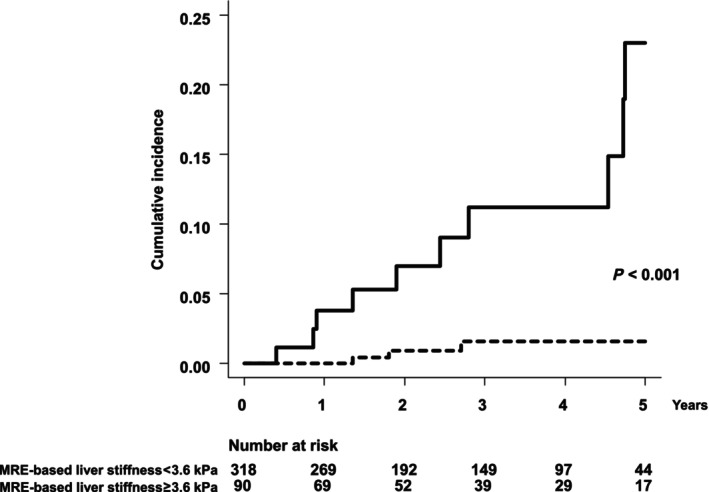
Cumulative incidence of HCC development. Patients were stratified by MRE‐based liver stiffness of 3.6 kPa, which indicates advanced fibrosis. HCC hepatocellular carcinoma; MRE, magnetic resonance elastography. 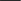, MRE‐based liver stiffness ≥3.6 kPa; 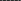, MRE‐based liver stiffness <3.6 kPa.

A total of 407 patients without HCC at the time of MRE were followed up with a mean of 2.9 years. During follow‐up, HCC developed in 13 patients. Patients were stratified by liver stiffness of 3.6 kPa, which was the threshold of advanced fibrosis. Of these, 12 patients were diagnosed with HCC with a single nodule and one patient (liver stiffness of ≥3.6 kPa) was diagnosed with HCC with three nodules. Ten patients developed HCC in patients with liver stiffness of ≥3.6 kPa and three patients developed HCC in patients with liver stiffness of <3.6 kPa. The 1‐, 3‐, and 5‐year cumulative incidence rates of HCC in patients with liver stiffness of ≥3.6 kPa were 3.8%, 7.0%, and 22.9%, respectively (Fig. 3). Similarly, the 1‐, 3‐, and 5‐year cumulative incidence rates of HCC in patients with liver stiffness of <3.6 kPa were 0%, 0.9%, and 1.5%, respectively. The cumulative incidence of HCC was significantly higher in patients with liver stiffness of ≥3.6 kPa than in those with liver stiffness of <3.6 kPa (*P* < 0.001). Multivariable analysis revealed that MRE‐based liver stiffness (per 1 kPa) was an independent factor for HCC development with an adjusted hazard ratio (aHR) of 1.61 (95% CI, 1.3–2.0, *P* < 0.001, Table 3). Similarly, compared with liver stiffness of <3.6 kPa, liver stiffness of ≥3.6 kPa was also an independent factor for HCC development with an HR of 8.22 (95% CI, 2.1–31, *P* = 0.002). Other factors including age, males, DM, dyslipidemia, and NA therapy were not associated with HCC development in the both analyses.

**Table 3:** MRE‐based liver stiffness and HCC development risk

	Hazard ratio	95% CI	*P* value
Univariable analysis			
MRE‐based liver stiffness per 1 kPa	1.69	1.4–2.0	<0.001
MRE‐based liver stiffness ≥3.6 kPa	11.25	3.1–40	<0.001
Multivariable analysis			
MRE‐based liver stiffness per 1 kPa	1.61	1.3–2.0	<0.001
Age	1.06	0.99–1.1	0.06
Males	2.38	0.6–9.5	0.2
DM	0.8	0.1–4.5	0.8
Dyslipidemia	0.36	0.07–1.9	0.2
NA therapy	2.61	0.7–10	0.2
MRE‐based liver stiffness ≥3.6 kPa	8.22	2.1–31	0.002
Age	1.06	0.99–1.1	0.05
Males	2.12	0.5–8.4	0.3
DM	1.1	0.2–6.1	0.9
Dyslipidemia	0.37	0.07–1.8	0.2
NA therapy	2.62	0.7–10	0.2

In the multivariable analysis, age, sex, DM, dyslipidemia, and NA treatment were adjusted.

CI, confidence interval; DM, diabetes mellitus; HCC, hepatocellular carcinoma; MRE, magnetic resonance elastography; NA, nucleoside/nucleotide analog.

## Discussion

### Main findings

This study demonstrated that MRE‐based liver stiffness is an HCC‐associated factor in patients with chronic hepatitis B in both cross‐sectional and longitudinal studies. Therefore, MRE may be used for fibrosis assessment, determination of indications for NA therapy, and early prediction of HCC development in patients with chronic hepatitis B.

### In context with published literature

Liver fibrosis is the most important factor associated with HCC development and prognosis in patients with chronic hepatitis B and other chronic hepatitis.^20^, ^21^, ^22^ Therefore, accurate evaluation of liver fibrosis is crucial in clinical practice. Several noninvasive modalities for liver fibrosis assessment have been developed.^23^, ^24^, ^25^ Since MRE has a higher diagnostic accuracy for liver fibrosis among these modalities, MRE is recommended as a screening and diagnostic modality for liver fibrosis in chronic hepatitis.^26^, ^27^, ^28^ Furthermore, in clinical trials of nonalcoholic fatty liver disease, MRE is used as a primary endpoint assessment modality instead of liver biopsy.^29^ Regarding MRE and HCC, previous studies have demonstrated that MRE‐based liver stiffness is associated with HCC development in patients with chronic liver disease or chronic hepatitis C.^16^, ^30^ However, data associated with MRE and HCC are limited, particularly in patients with chronic hepatitis B. A previous study including 180 patients with chronic hepatitis B demonstrated that MRE‐based liver stiffness is associated with HCC recurrence after hepatectomy.^31^ In this study, 504 patients with chronic hepatitis B were included, and a significant association was found between MRE and HCC. Our results support the clinical utility of MRE in patients with chronic hepatitis B, particularly the prediction of HCC development.

### Strengths and limitations

In this study, a relatively large number of patients whose MRE was measured were included. All patients received regular HCC surveillance with the aligned protocol. However, this is a retrospective, single‐center study; therefore, a further prospective, multicenter study is needed to establish the utility of MRE as a predictive factor for HCC development.

### Future implications

This study demonstrated that MRE is significantly associated with HCC development. In clinical practice, early detection of HCC development is important in patients with chronic liver disease. Therefore, MRE could be used as the prediction tool for HCC development.

In chronic hepatitis B, NA therapy is widely used and could reduce the risk of HCC development.^32^, ^33^ NA therapy is determined by alanine aminotransferase (ALT) and HBV‐DNA levels. However, several guidelines recommend that NA therapy should be initiated in patients at high risk of HCC, even if they are outside the ALT and HBV‐DNA criteria.^34^, ^35^ The effectiveness of NA therapy in reducing HCC risk is reduced in patients with cirrhosis. Therefore, early detection and introduction of NA therapy in patients at high risk of HCC is crucial for HCC deterrence.^36^, ^37^ MRE correlates with HCC risk and could be used to determine NA therapy indications.

Although HCC surveillance in patients at high risk of HCC is also an important clinical issue, it remains unclear how to identify patients at high risk of HCC. Since MRE is associated with the risk of HCC, MRE is useful in identifying patients requiring HCC surveillance. However, a few patients developed HCC even with liver stiffness of <3.6 kPa. Therefore, although MRE could identify advanced fibrosis and patients at high risk of HCC, identifying cases of HCC without fibrosis is a future challenge. HBV integration into host DNA causes carcinogenesis and such patients develop HCC without advanced fibrosis or cirrhosis.^38^ HBV markers (HBV‐DNA, HBsAg, HBcrAg, etc.) are associated with HCC development. In this study, HBsAg levels were significantly lower in patients with HCC than those without HCC. HBsAg levels decrease with age, and low HBsAg levels are associated with HCC risk in aging countries such as Japan, where HCC is more common in the elderly.^39^, ^40^ Other HBV markers are also associated with HCC risk.^41^, ^42^ Therefore, identifying cases of high risk by combining MRE and HBV markers is also a future challenge.

In conclusion, MRE‐based liver stiffness is associated with HCC risk in patients with chronic hepatitis B and may be used for the early prediction of HCC development and determination of indications for treatment.
